# Protective Effect of Selegiline (*R*-deprenyl) in Aminoglycoside-Induced Hearing Loss

**DOI:** 10.1007/s11064-025-04446-3

**Published:** 2025-06-13

**Authors:** Viktória Humli, Judit Szepesy, Gabriella Zsilla, Ildikó Miklya, Júlia Timár, Szilárd I. Szabó, Gábor Polony, Anita Gáborján, György B. Halmos, Petra Dunkel, Péter Mátyus, Balázs Lendvai, E. Sylvester Vizi, Tibor Zelles

**Affiliations:** 1 Department of Oral Biology, Semmelweis University, Budapest, Hungary; 2 Laboratory of Molecular Pharmacology, HUN-REN Institute of Experimental Medicine, Budapest, Hungary; 3 Department of Pharmacology and Pharmacotherapy, Semmelweis University, Budapest, Hungary; 4 Division of Neuroradiology, Vascular and Interventional Radiology, Department of Radiology, Medical University of Graz, Graz, Austria; 5 Department of Otorhinolaryngology, Head and Neck Surgery, Semmelweis University, Budapest, Hungary; 6 Department of Otorhinolaryngology, Head and Neck Surgery, University of Groningen, Groningen, The Netherlands; 7 Department of Organic Chemistry, Semmelweis University, Budapest, Hungary; 8 Present Address: National Laboratory of Infectious Animal Diseases, Antimicrobial Resistance, Veterinary Public Health and Food Chain Safety, University of Veterinary Medicine, Budapest, Hungary; 9 Pharmacology and Drug Safety Research, Gedeon Richter Plc, Budapest, Hungary

**Keywords:** Selegiline, Aminoglycoside-induced hearing loss, Dopamine, Ototoxicity, LOC, SNHLs

## Abstract

Aminoglycoside antibiotics remain indispensable despite their ototoxicity. Like other sensorineural forms, aminoglycoside-induced hearing loss (AGIHL) has no effective pharmacotherapy. Oxidative stress, apoptosis, excitotoxicity and inflammation are key pathological factors of the disease. We hypothesised that selegiline, an irreversible monoamine oxidase-B (MAO-B) inhibitor used in Parkinson’s disease, could be repurposed as an otoprotective agent against AGIHL and its effect on dopamine (DA) release from lateral olivocochlear (LOC) fibres, the efferent division of a protective feedback loop plays a major role in the protection against excitotoxicity. Selegiline mitigated AGIHL in BALB/c mice in a dose-dependent manner at different auditory brainstem response frequencies, including 16 kHz, the hearing sensitivity optimum of the animals. It also enhanced the action potential-evoked DA release from LOC efferents in mouse cochlear preparation dose-dependently. Inhibition of DA reuptake contributed to its basic effect of saving DA from metabolism. Among four selegiline analogues tested, the one that increased LOC DA release also provided otoprotection. In contrast, neither safinamide (a reversible MAO-B inhibitor) nor LJP-1207 (a selective semicarbazide-sensitive amine oxidase/vascular-adhesion protein 1 (SSAO/VAP1) inhibitor) prevented AGIHL, despite their antioxidant and anti-inflammatory properties. The reversibility or lack of MAO-B inhibition in safinamide and LJP-1207, respectively, as well as the absence of the propargylamine moiety with known intrinsic neuroprotective activity in both molecules, may explain their ineffectiveness. Selegiline, or certain propargylamine analogues of it, offer a promising therapy against AGIHL by addressing its multifactorial pathology through antioxidant, antiapoptotic, neuroprotective, and anti-inflammatory actions, while enhancing endogenous DAergic protective mechanisms.

## Introduction

Hearing loss (HL) is one of the most prevalent sensory impairments. Over 5% of the world’s population (~ 400 million adults and 34 million children) require rehabilitation for their disabling HL [1]. Contrary to conductive HLs, which can be treated with reconstructive middle ear surgery or hearing aids, there is no specific pharmacological treatment for the different types of sensorineural hearing losses (SNHLs). Age-related, noise- and ototoxic drugs-induced HLs are the most common forms of SNHLs.

Despite their potential ototoxicity, aminoglycoside antibiotics (AGs) remained important and in many cases indispensable medicines in the treatment of infections caused by aerobic gram-negative bacteria. High susceptibility of these bacteria to AGs, low levels of the development of antibacterial resistance and their cost-effectiveness are in favour of their use [2, 3]. Their affordability in combination with the high prevalence of tuberculosis in developing countries makes them among the most commonly used antibiotics worldwide [4]. However, 2–25% of patients suffer from SNHL after AG treatment [5].

AGs damage different cell types of the cochlea, including the inner and outer hair cells (IHCs, OHCs) and supporting cells in the organ of Corti and also the spiral ganglion cells (SGCs) [6–11]. The main pathological factors underlying AG-induced HL (AGIHL) are oxidative stress with reactive oxygen species (ROS) generation [12–14], apoptosis [15], excitotoxic damage of the auditory neurons [7, 16], and their synaptic contact with the HCs [17] and increased inflammatory responses [18].

**Fig. 1:**
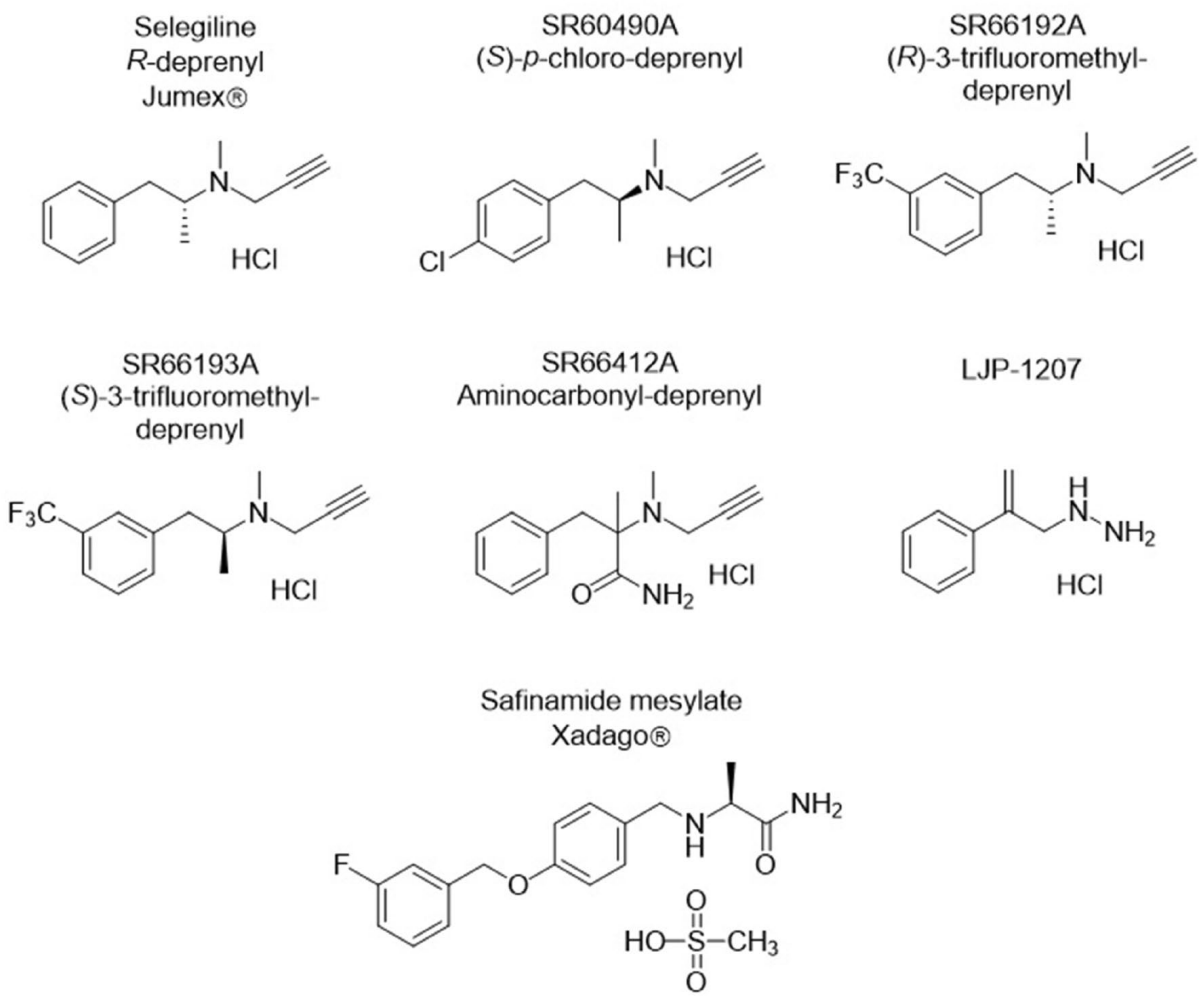
The chemical structure of selegiline (*R-*Deprenyl; Methyl-((*R*)-1-methyl-2-phenyl–ethyl)-prop-2-ynyl-amine hydrochloride), its four propargylamine analogues SR60490A ([(*S*)-2-(4-Chloro-phenyl)-1-methyl-ethyl]-methyl-prop-2-ynyl-amine; hydrochloride), SR66192A (Methyl-[(*R*)-1-methyl-2-(3-trifluoromethyl-phenyl)-ethyl]-prop-2-ynyl-amine; hydrochloride), SR66193A (Methyl-[(*S*)-1-methyl-2-(3-trifluoromethyl-phenyl)-ethyl]-prop-2-ynyl-amine; hydrochloride), SR66412A (2-Methyl-2-(methyl-prop-2-ynyl-amino)-3-phenyl-propionamide; hydrochloride) and the non-propargylamine LJP-1207 (2-phenylprop-2-enylhydrazine; hydrochloride) and safinamide mesylate ((2*S*)-2-[[4-[(3-fluorophenyl)methoxy]phenyl]methylamino]propanamide; methanesulfonic acid). Selegiline and safinamide are approved drugs for Parkinson’s disease under the brand names of Jumex® (Eldepryl) and Xadago®, respectively

Finding therapeutic compounds with protective effect in SNHLs, including AGIHL, is an unmet medical need. Development of new conventional small molecules or biological products, including gene therapy, is a promising direction in drug research for SNHLs [19], as well, but repositioning provides a drug discovery strategy with significant advantages, including reduced financial investment, substantially shortened drug development timelines and potentially higher success rates. Several drugs registered for use in neurodegenerative diseases target molecular and cellular mechanisms involved in the development of AGIHLs. Selegiline, also known as *R-*deprenyl (Jumex®; Fig. 1), is a registered, irreversible and selective monoamine oxidase B (MAO-B) enzyme inhibitor [20] found to be effective and well-tolerated in the treatment of Parkinson’s disease (PD) and depression [21–23]. By inhibiting its degradation via MAO-B, it enhances the level of DA, which causes a symptomatic improvement in PD. Moreover, it has several additional beneficial properties, including anti-apoptotic [24], antioxidant [25], neuroprotective [26] and immunomodulatory [27] effects, which are considered responsible for its disease-modifying action [22, 28].

The increased DAergic neurotransmission can result in another otoprotective effect of selegiline. In numerous forms of SNHL, including AGIHL, an excessive amount of glutamate (Glu) is released from IHCs and initiates excitotoxic damage of the synapsing primary auditory neurons, the SGCs [7, 29, 30]. Harmful overactivation of SGCs is attenuated by DA released from the lateral olivocochlear (LOC) efferents, forming axodendritic synapses on the peripheral dendrite of the type I SGCs [29, 31–33]. LOC fibres are the efferent division of the *SGCs—cochlear nucleus—lateral superior olivary complex—SGCs* short-loop feedback [34–37]. DA receptor agonists were shown to reduce the characteristic electrophysiological and structural changes in SGCs evoked by different acoustic trauma, ischemia or NMDA- and AMPA-enhanced firing [38–41]. Therefore, selegiline, with its boosting effect on LOC DAergic neurotransmission, is supposed to be otoprotective in AGIHL. Indeed, in our previous experiments, selegiline has shown a protective effect in presbycusis [35], and another selective MAO-B inhibitor, rasagiline, has also exerted otoprotection in our AGIHL mouse model [34]. Contrary to rasagiline, selegiline also has a monoaminergic activity enhancer effect through the trace amine-associated receptor 1 (TAAR1) [42]. These mechanisms underscore the otoprotective potential of selegiline in AGIHL and underline the rationale to examine that.

In order to better understand the mechanism of the otoprotective action of selegiline and to broaden the range of drugs that could potentially be repositioned, we have also investigated the protective effect of two other compounds. They are non-propargylamine derivatives but show some similarity in chemical structure and a partial overlap in pharmacodynamic action to selegiline.

Safinamide (Xadago®; Fig. 1) is a reversible MAO-B inhibitor approved by the U.S. Food and Drug Administration (FDA) as an anti-PD medication [43]. It protects neurons from Glu excitotoxicity [44] and shows some antioxidant and anti-inflammatory effects, as well [45, 46].

LJP-1207 (Fig. 1) is a representative member of semicarbazide-sensitive amine oxidase/vascular-adhesion protein 1 (SSAO/VAP1) enzyme inhibitors [47, 48]. The activity of the enzyme could induce oxidative stress [48, 49], apoptosis and inflammation [47, 49], therefore, SSAO inhibition was assumed to have a beneficial effect in AGIHL, too. Indeed, inhibition of its activity protected DA neurons by decreasing the production of ROS [50], and the application of LJP-1207 has been useful in the prevention or treatment of inflammatory disorders [48].

Our findings revealed that the multitargeted PD drug selegiline (Jumex®) has attenuated the AGIHL in a dose-dependent manner. Its multiple antioxidant, antiapoptotic, neuroprotective, anti-inflammatory and LOC DA release enhancer actions might be responsible for the protection in this disease with a complex, multifactorial pathomechanism. Two chemically similar, but non-propargylamine compounds, including safinamide (Xadago®), with overlapping, but not identical pharmacodynamic actions proved to be ineffective. These findings suggest that repositioning of certain registered drugs with a favourable combination of action on different protective modalities could be a feasible and time-saving way to introduce new drug therapy for AGIHL or, in a broader sense, for other SNHL forms.

## Materials and Methods

### Tested Compounds

The chemical structures and names of compounds tested in this study are shown in Fig. 1. Selegiline and its four analogues were synthesised by Chinoin Pharmaceutical and Chemical Works Private Co. Ltd., Budapest, Hungary; LJP-1207 and safinamide mesylate at the Department of Organic Chemistry, Semmelweis University, Budapest, Hungary.

### Animals and Housing Conditions

The in vitro [^3^H]DA release experiments were conducted on male CD-1 mice (20–35 g) bred at the Medical Gene Technology Unit of HUN-REN Institute of Experimental Medicine. The in vivo experiments were carried out on four-week-old male BALB/c mice (*BALB/cAnNCrl*) ordered from Charles River Laboratories, Germany. The experiments started after 3 days of acclimatisation. The animals were housed under a normal day/night cycle (L-D/12–12), controlled temperature (22 ± 2 °C) and humidity (40–60%) with food (standard laboratory chow) and water provided *ad libitum*.

### In Vitro Measurement of [^3^H]DA Release from the LOC Terminals in the Mouse Cochlea

The in vitro superfusion technique was used as we described earlier [31, 34, 51]. In summary, mice were anesthetised superficially by isoflurane and then decapitated. The cochleae were removed and placed immediately in a perilymph-like solution (150 mM NaCl, 3.5 mM KCl, 1 mM CaCl_2_, 1 mM MgCl_2_, 2.75 mM Hepes and 2.25 mM Tris. The pH was adjusted to 7.4. The osmolarity was set by D-glucose, and the solution was gassed continuously with 100% O_2_. Under stereomicroscopic guidance, the bony capsule of the cochlea, together with the spiral ligament and stria vascularis, were chipped away, and the modiolus was fractured at the base of the cochlea. The preparation contains the spiral ganglion, the afferent auditory fibres, the axons and axon terminals of the efferent bundles and the organ of Corti with the IHC and OHC. The viability of the cochlear preparation was shown by light- and electron microscopy performed immediately before and after the experiments [31].

The dissected cochleae were incubated with 0.2 µM [^3^H]DA (specific activity: 48–66 Ci/mmol; [7,8-^3^H]DA, Amersham, UK) for 35 min then placed into a microvolume plexi chamber (three cochleae per chamber) and superfused with the perilymph-like solution (3 ml/min). Following a one-hour pre-perfusion period, the effluent was collected in three-minute fractions. The radioactivity in the fractions, indicating the release of [^3^H]DA from the LOC terminals, was quantified by analysing 500 µl aliquots of each sample using a liquid scintillation counter (Ultima Gold™ Liquid Scintillation Cocktail; Packard Tri-Carb 1900TR). Following the 57-min collection of the samples (19 fractions), the cochleae were transferred to 500 µl of 10% trichloroacetic acid for one day. Thereafter, 100 µl was used to measure the tissue content of radioactivity. Our HPLC measurements demonstrated that 91–95% of the released radioactivity was attributable to [^3^H]DA and its metabolites, 3,4-Dihydroxyphenylacetic acid and Homovanillic acid [52]. To evoke action potentials in LOC efferents, electrical field stimulations (30 V, 5 Hz, 0.5 ms impulse duration, for 3 min) was applied in the 3rd (S_1_) and 13th (S_2_) fractions by a Grass S88 stimulator (Grass Instrument Co., Quincy, MA) via platinum electrodes positioned at the upper and lower parts of the tissue chamber.

Selegiline and its analogues (Fig. 1) were added to the perfusion at the beginning of the 8th fraction (21st min) and maintained until the end of the experiment. Perfusion of CdCl_2_ and tetrodotoxin (TTX) was started 6 min earlier (from the 15th minute). Nomifensine application and temperature reduction to 17 °C were started at the 45th minute of pre-perfusion and maintained until the end of the experiment. The fractional release (FR) of tritium was calculated as a percentage of the total radioactivity present in the tissue at the time of sample collection [53, 54]. The FR due to the field stimulations (S_1_ and S_2_) was calculated by subtracting the mean of the basal release, determined from FR values before and after the stimulation, from the total FR during the electrical stimulation [32, 55]. This calculation is referred to as the area under the curve. The impact of pharmacological agents on field stimulation-evoked [^3^H]DA release was quantified by calculating the ratio of FRS_2_ to FRS_1_ (FRS_2_/FRS_1_).

The reversibility and reproducibility of the field stimulation-evoked response and its inhibition by VGSC or VGCC blockade confirm that the applied field stimulations induce action potential-triggered, extracellular Ca^2+^-dependent vesicular exocytosis of [^3^H]DA [31, 51, 52].

### Mouse Model of Aminoglycoside-Induced Hearing Loss (AGIHL) for Testing Otoprotective Effect In Vivo

To test the protective effect of different compounds in aminoglycoside-induced hearing loss (AGIHL), we used an in vivo mouse model installed in our laboratory [34], based on Wu et al. [12]. The mouse is a well-established subject for modelling human SNHLs, because of the similar cochlear anatomy, physiology and pattern of ototoxicity-related hearing loss, like earlier and the more severe hearing impairment at the higher frequencies in aminoglycoside-induced hearing loss (AGIHL) [12, 56, 57].

#### Assessing the Hearing Function of Mice by Measuring the Auditory Brainstem Responses (ABRs)

**Fig. 2:**
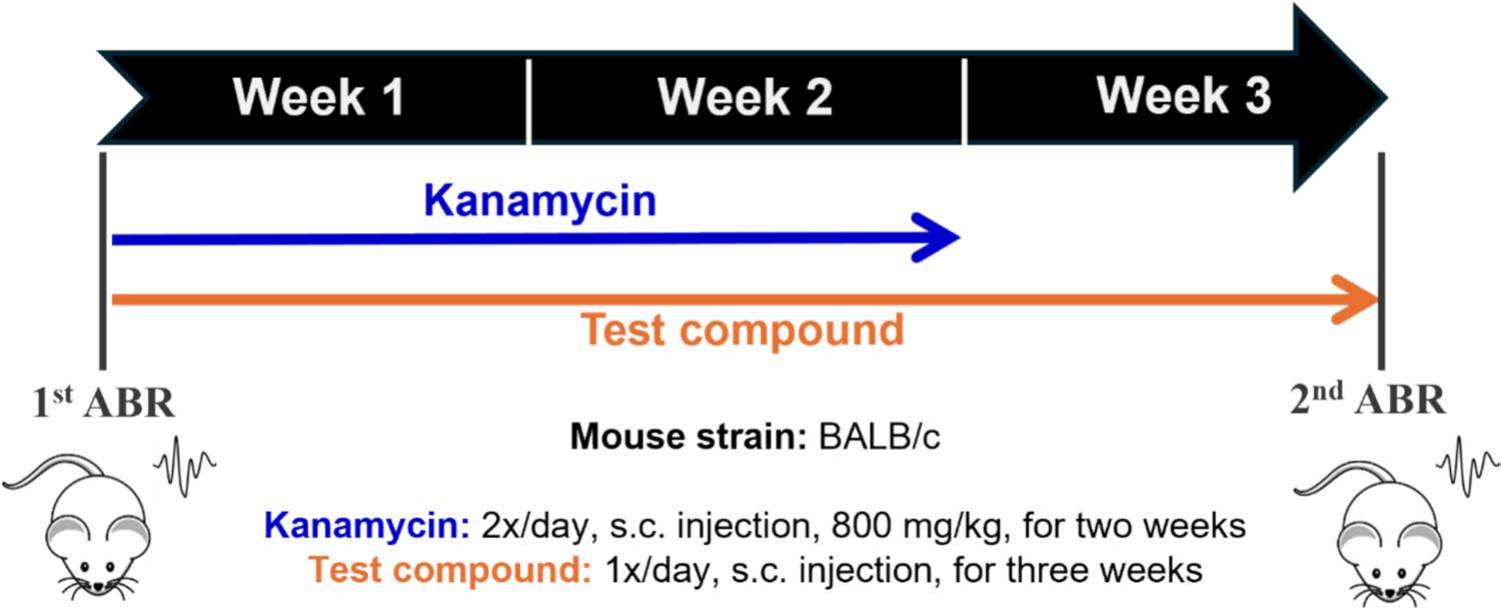
Experimental design for testing the otoprotective effect of compounds in the kanamycin-induced hearing loss mouse model (an AGIHL model). Kanamycin (800 mg/kg, s.c.) was administered twice daily for two weeks to induce hearing loss, while the test compounds were administered once daily (s.c.) for three weeks. ABR measurements were performed before (1st ABR) and after (2nd ABR) the treatment period to calculate the auditory threshold shifts

ABR measurements were carried out to evaluate the auditory function. In brief, the mice were anaesthetised by intraperitoneal (i.p.) injections of ketamine (100 mg/kg, CP-Ketamin 10% injection, Produlab Pharma B.V., The Netherlands) and xylazine (10 mg/kg, CP-Xylazine 2% injection, Produlab Pharma B.V., The Netherlands). The body temperature of the mice was maintained at 37 °C throughout the ABR measurement via a feedback-controlled heating pad (Supertech Physiological and Biological Temperature Controller TMP-5b). To prevent corneal desiccation, ophthalmic gel (Corneregel, Baush & Lomb) was applied to both eyes of the mice. Auditory thresholds were obtained using an ABR workstation from TDT (System3, Tucker-Davis Technologies, Alachua, FL). To elicit ABR responses, click stimuli (0.4 ms duration) and tone bursts (3 ms duration, 0.2 ms rise/decay; 4–8–16 kHz) were generated by the TDT SigGen software package and delivered to the right external ear canal in a closed acoustic system via a plastic tube connected to an EC1 electrostatic loudspeaker. For system calibration, a measurement microphone (model 4016) produced by ACO Pacific Inc. was used (Belmont, CA, USA). ABR waves were recorded via subdermal electrodes (Rochester Electro-Medical, Inc.; Lutz, FL) placed subcutaneously (s.c.) at the vertex (active electrode), behind the right ear (reference electrode) and at the hind leg (ground). Sound intensity was increased in 10 dB steps from 0 to 80 dB in click stimulation mode. Intensity of tone burst stimuli was attenuated in 10 dB steps from 90 to 10 dB at each measured frequency. Hearing threshold was defined as the lowest intensity level at which an ABR wave with an identifiable peak could be observed. ABR waves were recorded in each animal before starting the drug treatments (baseline hearing threshold), then 3 weeks later at the end of the experiment (Fig. 2). The auditory threshold shift is defined as the difference between the second and first auditory threshold value.

#### Drug Administration and Experimental Design

All drugs were dissolved in saline (SAL). Kanamycin sulphate (ultra pure, USB Corporation, Cleveland, OH/Affymetrix, Santa Clara, CA) was administered s.c. into the dorsal region of the animals behind the neck twice daily for two weeks at a dose of 800 mg/kg [12, 34]. The investigated drugs (selegiline, SR60490A, LJP-1207 and safinamide) were administered once daily (s.c.) at the same time as the first kanamycin dose of the day, but in a separate injection. Their administration lasted 3 weeks (Fig. 2).

The arrangement of the experiments was as follows. Mice were randomised and 20 of them were assigned to each experimental group. (1) Control (SAL), (2) Kanamycin (800 mg/kg), (3) Kanamycin (800 mg/kg) plus selegiline or SR60490A (0,5 mg/kg), (4) Kanamycin (800 mg/kg) plus selegiline or SR60490A (3 mg/kg), (5) Kanamycin (800 mg/kg) plus selegiline or SR60490A (6 mg/kg each), (6) selegiline or SR60490A alone (6 mg/kg each). Because of the limited number of ABR measurements that can be performed in a day, for testing the effect of selegiline and SR60490A in three different doses with kanamycin plus alone in the highest 6 mg/kg dose, the experiments had to be carried out in three different runs with repeated testing of kanamycin and control (SAL). In a separate set of experiments, we also tested the effect of the SSAO inhibitor LJP-1207 and the reversible MAO-B inhibitor safinamide in the following arrangement: (1) Control (SAL), (2) Kanamycin (800 mg/kg), (3) Kanamycin (800 mg/kg) plus LJP-1207 (20 mg/kg), (4) Kanamycin (800 mg/kg) plus safinamide (10 mg/kg). The reduction in sample size for certain control and treatment groups was due to unforeseen factors, including unexpected mouse mortality, ear canal damage, or technical errors during ABR measurements.

### Behavioural Testing

The effect of selegiline, in the highest dose administered in the AGIHL model, was assessed on anxiety-like behaviours and general motor activity in the BALB/c mice.

#### Elevated Plus-Maze Test (EPM)

The apparatus consisted of two open arms (50 × 15 cm) and two closed arms (50 × 15 cm, with a 40 cm surrounding wall) extending from a central platform (15 × 15 cm) and positioned in opposition to one another. The maze is elevated 70 cm above the floor and placed in an isolated, dark room. The arms are illuminated. Mice were placed into the centre of the apparatus, and their movements were recorded and subsequently analysed by an experimenter who was blind to the treatments using a computer-based event recorder (H77, Budapest, Hungary). The observation lasted 5 min, and the time spent in the open and closed arms was recorded.

#### Locomotor Activity Test

Locomotor activity (LA) was quantified using the CONDUCTA System for behavioural and activity studies (Experimetria Ltd, Budapest, Hungary). The apparatus comprises three black-painted testing boxes (40 × 50 × 50 cm each) placed in an isolated room. The movements of the mice were recorded utilising high-density arrays of infrared diodes. One animal was placed in each of the three boxes, which can be tested simultaneously by the apparatus. The animals were not connected to each other. The time spent ambulating (i.e., walking or running) was recorded for each individual box. The observation started immediately without any habituation and lasted 40 min.

### Statistical Analysis

The data from the [^3^H]DA release experiments are presented as means ± standard error of the mean (SEM). Statistical analysis was performed using one-way ANOVA, followed by the Bonferroni post-hoc test, in GraphPad Prism 6 (GraphPad Software, San Diego, CA, USA). In the AGIHL model experiments, individual auditory threshold shifts (in decibel sound pressure level, dB SPL) were determined for each mouse, and subsequently, group averages ± SEM were calculated. These data were analysed using two-way ANOVA, followed by the Bonferroni post-hoc test in GraphPad Prism 6. For the behavioural tests, data are also presented as means ± SEM, with statistical significance evaluated using one-way ANOVA, which was also applied to analyse weight gain changes. In all analyses, the following significance thresholds were used: *p < 0.05, **p < 0.01, ***p < 0.001, ****p < 0.0001, and ##*p* < 0.01, ####*p* < 0.0001.

## Results

### Selegiline Enhanced the Electrical Stimulation-Evoked Release of DA from LOC Terminals Dose-Dependently in the Mouse Cochlea

**Fig. 3:**
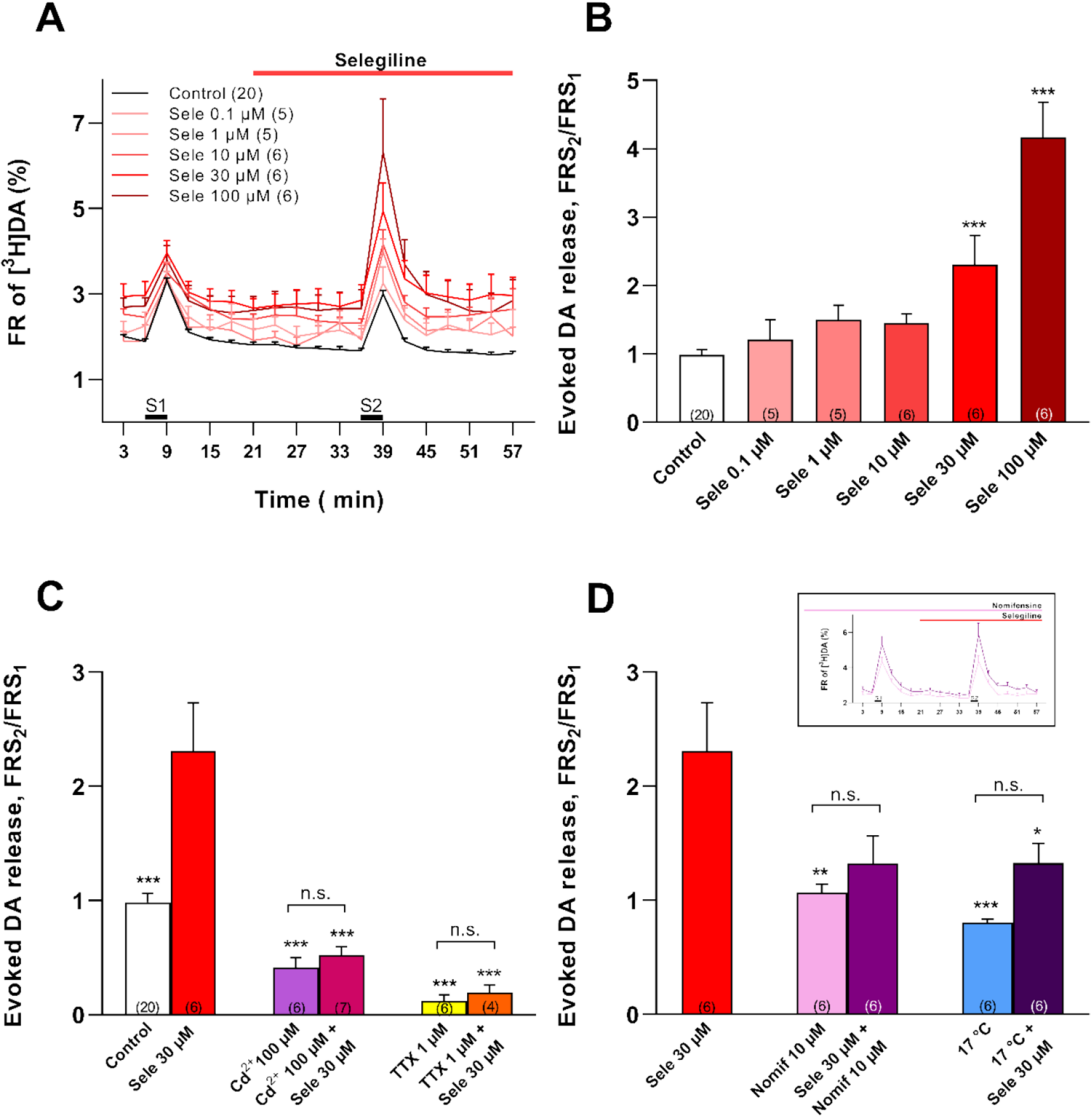
Selegiline increased the electric stimulation-evoked release of [^3^H]DA from LOC terminals in the isolated mouse cochlea preparation dose-dependently. The effect of selegiline was dependent on the function of the voltage-gated Ca^2+^ (VGCCs) and Na^+^ channels (VGSCs) and on the reuptake of DA. **A** The graph shows the selegiline (Sele) effect on the electrical stimulation-evoked release of [^3^H]DA. The time of selegiline perfusion (0.1–100 µM) is indicated by the red horizontal line. Control trace (black line) means no selegiline perfusion. Different shades of red lines show the effect of different concentrations of selegiline. S_1_ and S_2_ bars show the electrical field stimulations. **B** The bar graph illustrates the impact of the different selegiline concentrations on the electrical stimulation-evoked DA release (FRS_2_/FRS_1_). The asterisks indicate the significant differences compared to the Control. **C** The blockade of VGCCs with Cd^2+^ (CdCl_2_, 100 µM) and of VGSCs with tetrodotoxin (TTX, 1 µM) inhibited the effect of electrical stimulation and prevented the potentiating effect of selegiline. The asterisks indicate the significant differences compared to the effect of selegiline (30 µM). The differences of Cd^2+^ vs. Cd^2+^ + selegiline and TTX vs. TTX + selegiline were not significant (n.s.). **D** Reuptake inhibition by nomifensine (Nomif, 10 µM; Inset) perfusion and lowering the temperature to 17 °C was started 15 min prior to the commencement of DA release measurements and was maintained throughout the experiment. Both interventions decreased the effect of selegiline (30 µM) on stimulation-evoked release of [^3^H]DA significantly. The asterisks indicate the significant differences compared to the effect of 30 µM selegiline. In the presence of nomifensine and at 17 °C, selegiline (30 µM) could not enhance the stimulation-evoked [^3^H]DA release significantly (n.s.). Data are presented as mean ± SEM; *p < 0.05, **p < 0.01, ***p < 0.001, n.s. = not significant; One-way ANOVA followed by Bonferroni post-hoc test. Numbers in parentheses indicate the number of independent experiments (n)

Selegiline enhanced the electrical field stimulation (S_2_) evoked release of [^3^H]DA in the mouse cochlea preparation. Its effect showed dose-dependency (Fig. 3A, B) and suggested a modulatory effect on the action potential-evoked, Ca^2+^-dependent exocytotic release of DA from LOC terminals. Inhibition of the effect of 30 µM selegiline by blocking VGCCs with Cd^2+^ (100 µM), supported this notion (Fig. 3C). Elimination of selegiline effect by the blockade of the VGSCs (TTX, 1 µM) showed that axonal firing triggered the exocytotic release of DA, what was increased by selegiline (Fig. 3C). Besides inhibiting MAO, selegiline also inhibits the reuptake of DA to the nerve terminals [58] what can contribute to the augmentation of DA release by the drug. Inhibition of the uptake molecules from the 45th min of preperfusion (Fig. 3D, Inset) by the selective DA uptake inhibitor nomifensine (10 µM) [31, 34], or low temperature (17 °C) [34, 59, 60] suppressed the boosting effect of selegiline (30 µM) on the field stimulation-evoked release of [^3^H]DA (Fig. 3D).

### Testing the DA Releasing Effect of Selegiline Analogues

**Fig. 4:**
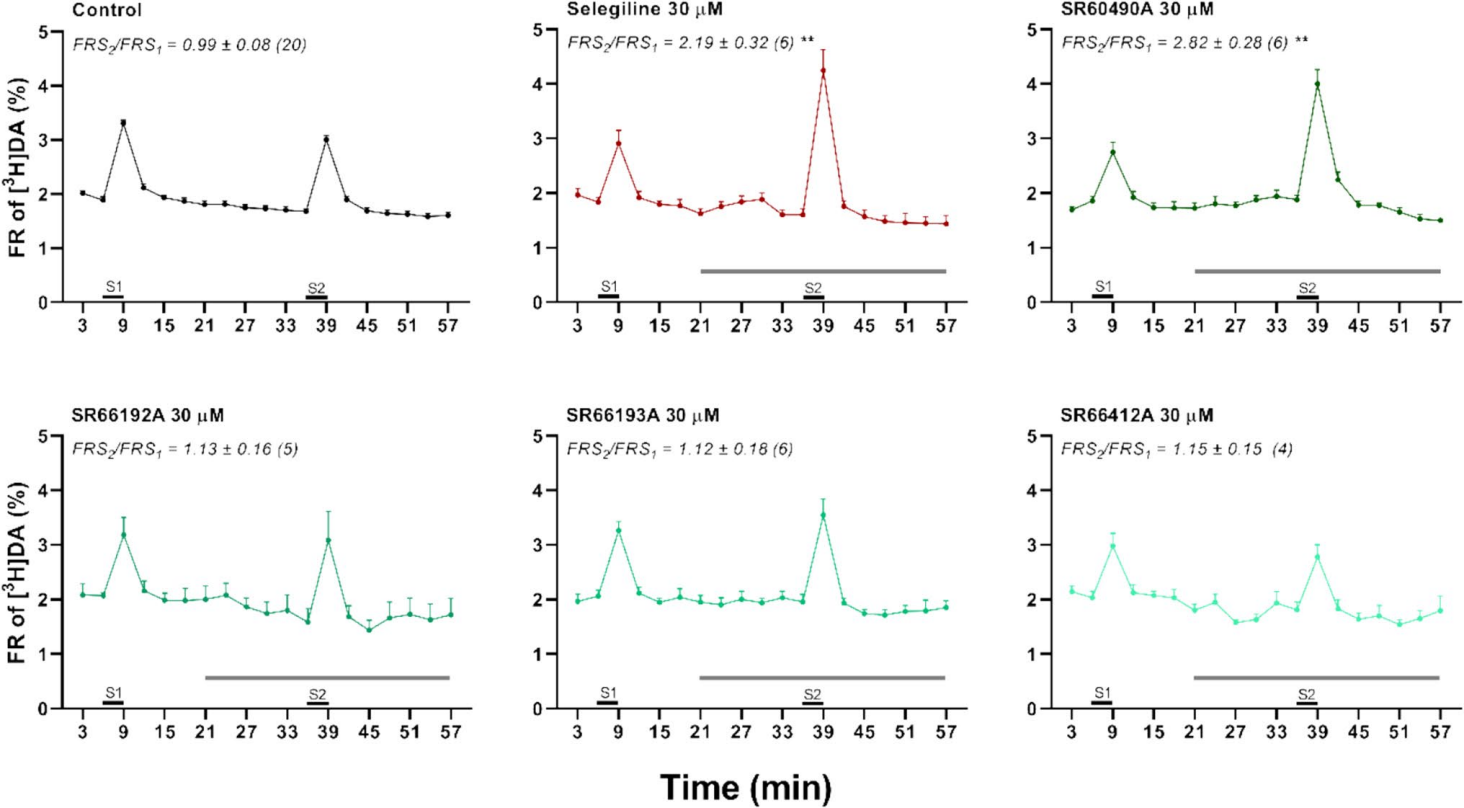
Effect of selegiline and its analogues on the release of [^3^H]DA from mouse cochlear preparation. Selegiline and SR60490A, but not the other selegiline-related compounds (SR66192A, SR66193A and SR66412A), potentiated significantly the field stimulation-evoked [^3^H]DA release. Electrical field stimuli are indicated by S_1_ and S_2_ (horizontal black bars). FRS_2_/FRS_1_ value of the actual compound was compared to the Control (**p < 0.01). Data are presented as mean ± SEM; One-way ANOVA followed by Bonferroni post-hoc test. The number of independent experiments is in parentheses (n)

Four analogues of selegiline with improved pharmacokinetic properties (SR60490A, SR66192A, SR66193A and SR66412A; Fig. 1) were also tested in 30 µM concentration on the release of [^3^H]DA from LOC terminals. One of those, the SR60490A, increased the electric stimulation-evoked release, similarly to selegiline. The other three compounds did not influence [^3^H]DA release (Fig. 4).

### Selegiline and (*S*)-*p*-Chloro-Deprenyl (SR60490A) Attenuated the Aminoglycoside Antibiotic-Induced Hearing Loss in BALB/c Mice

**Fig. 5:**
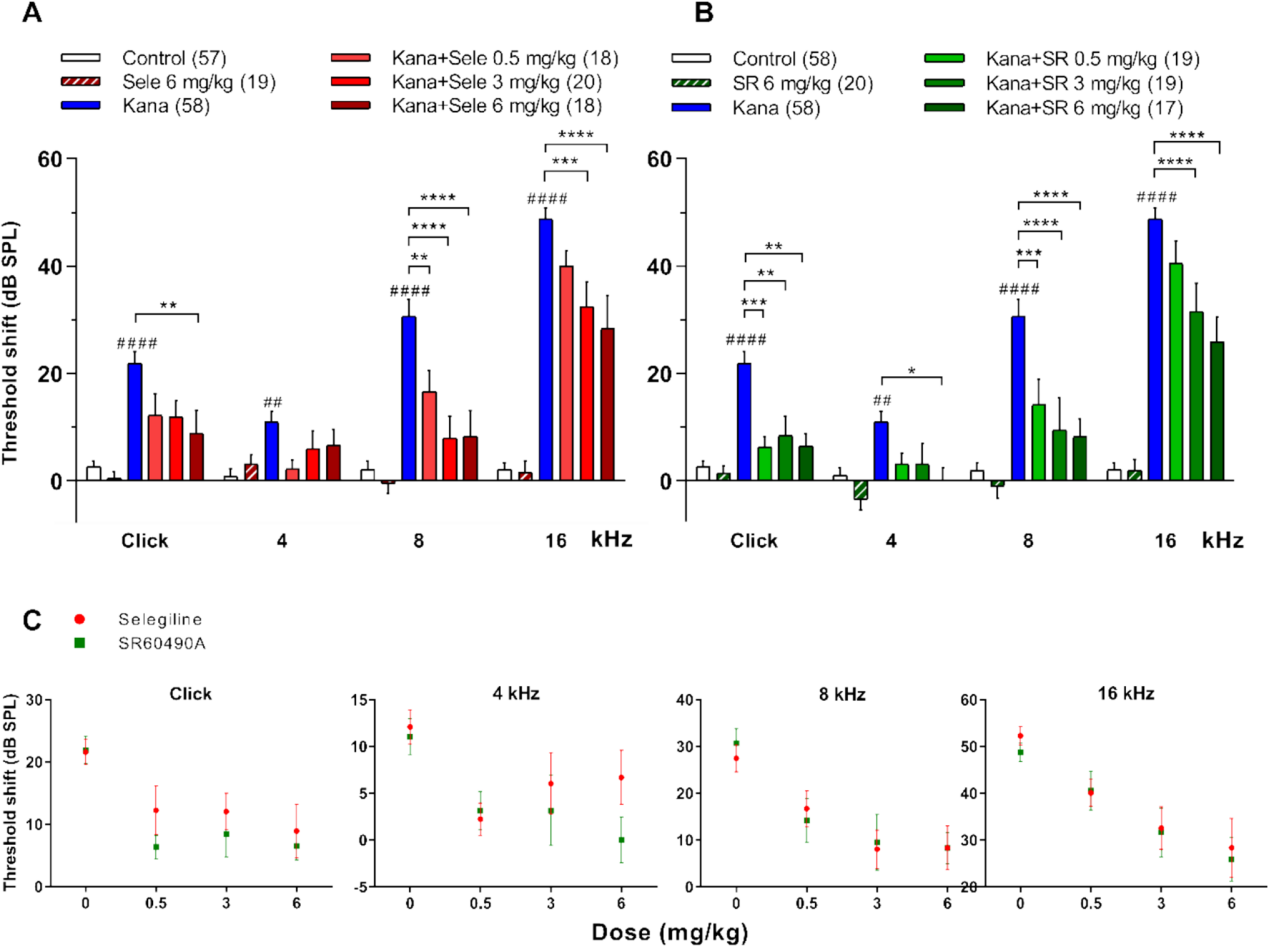
Selegiline and its analogue SR60490A attenuated the kanamycin-induced hearing impairment in BALB/c mice (AGIHL model). Kanamycin administration (800 mg/kg, s.c.) increased the auditory threshold significantly at all measured frequencies and click stimuli. The graphs show the shift in the auditory threshold at different frequencies and click stimuli during the 3-week-long experiments. Threshold shifts were determined in individual mice as the difference of threshold measured at the end of the experiment (2nd ABR) and right before the start of drug administrations (1st ABR; see Fig. 2). The protective effect appears as the reduction in the kanamycin-evoked auditory threshold shift. **A** Selegiline administration (0.5, 3 and 6 mg/kg, s.c.) mitigated the kanamycin-evoked threshold shift dose-dependently at different frequencies and click stimuli, while it had no effect alone, compared to the saline Control. **B** Application of SR60490A in the same doses (0.5, 3 and 6 mg/kg, s.c.) resulted in similar dose-dependent protective effects. Although it also had a significant effect at 4 kHz in its highest dose and at click stimuli in the doses of 0.5 and 3 mg/kg. **C** The difference between the protective effects of selegiline and SR60490A was not significant. Data are presented as the mean of individual threshold shifts in dB ± SEM; *p < 0.05, **p < 0.01, ***p < 0.001, ****p < 0.0001 versus kanamycin-treated group; ## *p* < 0.01, #### *p* < 0.0001 vs. Control. Numbers in parentheses indicate n values in the respective control or treatment groups (see Materials and Methods). The two-way ANOVA and Bonferroni post-hoc test were used to statistically analyse the data

The effect of selegiline and its analogue SR60490A (Fig. 1), which increased the field stimulation-evoked DA release from LOC terminals in the cochlea, was investigated at different frequencies and click stimuli in the AGIHL mouse model. The impairment of hearing by kanamycin (s.c.) reaches its plateau at 3 weeks [34], therefore the hearing function of mice was remeasured after 3 weeks (2nd ABR) of treatment by the two compounds (s.c.; Fig. 2). The increase in auditory threshold, compared to the corresponding one measured by ABR right before the onset of treatments (1st ABR), represents the auditory threshold shift, i.e., the hearing impairment, determined in mice individually. Kanamycin administration resulted in a significant hearing impairment at all measured frequencies and click stimuli (Fig. 5A and B). Selegiline, in the applied doses (0.5, 3 and 6 mg/kg, s.c.) significantly attenuated kanamycin-induced threshold shift in a dose-dependent manner. Its protective effect was observed at both click stimuli and at 8 and 16 kHz tone bursts, respectively. The highest used dose of selegiline (6 mg/kg) alone had no effect on hearing at any frequencies, similarly to the saline (s.c.) control (Fig. 5A). Administration of SR60490A in the same doses (0.5, 3 and 6 mg/kg, s.c.) produced protection against hearing loss at all the frequences tested and click stimuli. Protection by SR60490A demonstrated dose-dependency, similar to selegiline. Application of SR60490A alone at the highest dose (6 mg/kg) had no effect at any frequency (Fig. 5B). Otoprotective effect of SR60490A seemed to be more pronounced, as it was statistically significant also at 4 kHz in 6 mg/kg dose and 0.5 and 3 mg/kg at click stimuli. However, the protection by selegiline vs. SR60490A was not different statistically (Fig. 5C).

### An SSAO and a Reversible MAO-B Inhibitor with Non-propargylamine Chemical Structures Are Not Protective Against AGIHL

**Fig. 6:**
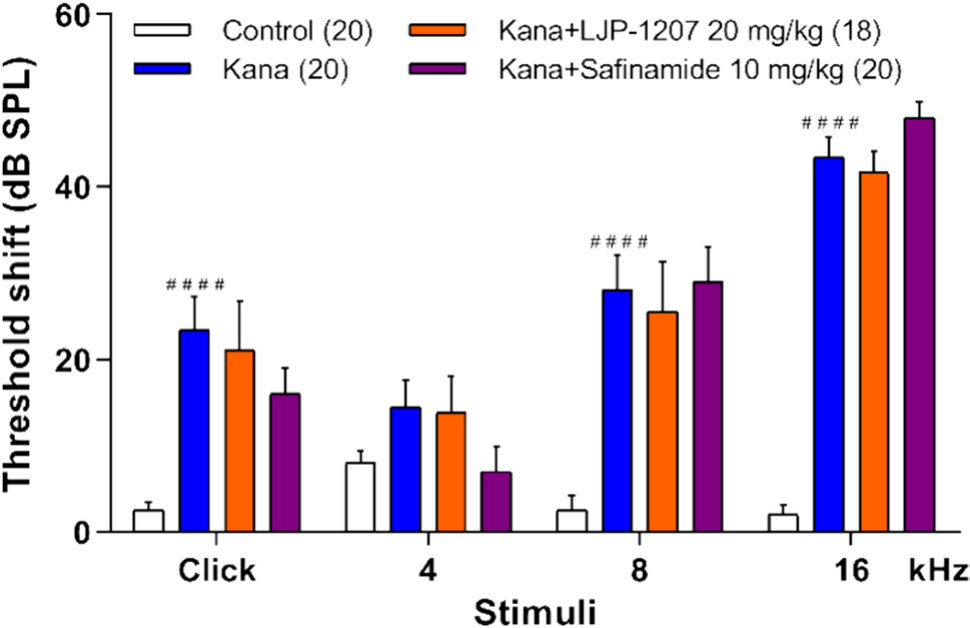
The SSAO inhibitor LJP-1207 and the reversible MAO-B inhibitor safinamide, both with non-propargylamine chemical structure, failed to mitigate the kanamycin-induced hearing loss (AGIHL). The graph shows the individual threshold shifts calculated at different frequencies and click stimuli as the difference of the auditory threshold at the end (2nd ABR) and the onset (1st ABR) of the 3-weeks long experiment (Fig. 2). Data are presented as the mean of individual threshold shifts in dB ± SEM. Two-way ANOVA did not reveal statistically significant differences. #### *p* < 0.0001 vs. Control. Numbers in parentheses indicate n values in the respective control or treatment groups

In a separate set of experiments, we investigated the effect of LJP-1207 (20 mg/kg, s.c.) and safinamide (10 mg/kg, s.c.), an amine oxidase and a reversible MAO-B inhibitor (Fig. 1), respectively, in the AGIHL mouse model. Administration of neither non-propargylamine compounds resulted in any protection against the AGIHL (Fig. 6).

### Selegiline Alone or in Combined Administration with Kanamycin Did Not Affect the Behaviour and Physiological Weight Gain of the Mice

At high doses of selegiline, (*R*)-methamphetamine and (*R*)-amphetamine, among the metabolites formed, may produce effects typical of amphetamine as a psychostimulant [22]. To test this possibility, we investigated whether selegiline alone in the highest dose we used (6 mg/kg) or in combination with kanamycin affects anxiety-like behaviour, general motor activity or age-related weight gain.

**Fig. 7:**
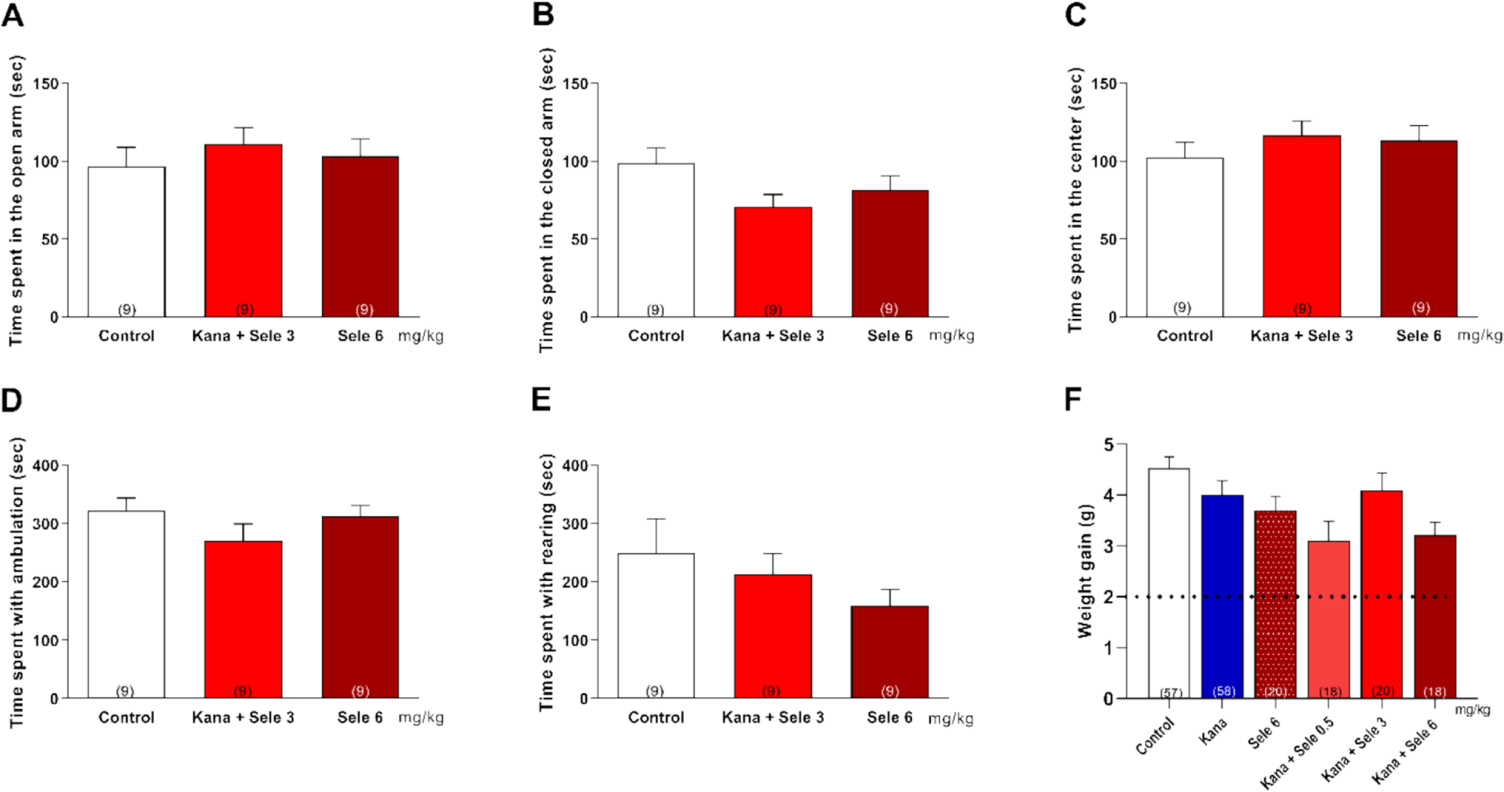
Selegiline and selegiline plus kanamycin treatment did not affect anxiety-like behaviour, general motor activity or age-related weight gain in BALB/c mice. The upper row of the graph shows the time mice spent **A** in the open arm, **B** in the closed arm and **C** in the centre of the elevated plus maze from the total observation period of 5 min during their Elevated plus-maze test. 3 weeks long administration (s.c.) of selegiline in the highest used 6 mg/kg dose or selegiline plus kanamycin treatment did not cause any anxiogenic effect. **D** and **E** The same treatments were without any effect on the locomotor activity of the mice. **F** Weight gain of mice during the 3-week-long experiment was in the normal physiological and age-dependent range of the BALB/c strain (dotted line, minimum species-specific, physiological weight gain during the experimental period), and treatment groups were not significantly different from the control one. Data are the mean ± SEM; the number of experiments is given in parentheses. The statistical significance was evaluated using one-way ANOVA

Elevated plus-maze test did not demonstrate any difference between control animals and selegiline or selegiline + kanamycin treated mice, suggesting that selegiline does not cause any anxiety-like behaviour in the highest 6 mg/kg dose we used (Fig. 7A–C). Quantification of locomotor activity in these mice revealed no statistically significant differences between the control and treatment groups (Fig. 7D and E). Weight gain of mice during the 3-week-long selegiline (Fig. 7F) and SR60490A (data not shown) experiment was also within the physiological and normal age-dependent range of the strain (Charles River Laboratories, https://www.criver.com/products-services/find-model/balbc-mouse?region=3631; Fig. 7F).

## Discussion

Hearing loss is the most frequent human sensory deficit, affecting millions of people worldwide. Its sensorineural forms lack effective pharmacotherapy available to prevent, cure or restore hearing function. The most prevalent causes of SNHLs are aging, noise exposure and the use of ototoxic medications. Recent advances have provided deeper insights into the molecular mechanisms of sensorineural hearing loss (SNHL) and the development of potential inner ear therapeutics.

Aminoglycoside antibiotics (AGs) are well known for their ototoxic effect. However, they remain indispensable for preventing and treating life-threatening infections [2, 3]. They are crucial in managing infections caused by aerobic, multidrug-resistant Gram-negative bacteria intra-abdominally, in cystic fibrosis, recurrent and resistant tuberculosis, severe cases of endocarditis or in both early-onset and late-onset neonatal sepsis [61]. In neonatal intensive care units (NICUs), all neonates routinely receive a preventive combination of gentamicin and ampicillin as part of standard care protocols. While the prevalence of HL in the general newborn population is estimated at 1 per 1,000 live births, the prevalence of HL among infants admitted to NICUs is almost seven times higher than for those not admitted. One major underlying cause of this is the exposure to ototoxic antibiotics [62]. Similar to other forms of SNHLs, excitotoxicity [7, 16], oxidative stress [14, 57, 63], apoptosis [13] and inflammation [14, 18, 64] play a crucial role in the development of AGIHL. Given the absence of effective pharmacotherapy, there remains an urgent need to find medications that can prevent or treat AGIHL. Considering that the protocol for administering AGs is well-known and predictable, there is an opportunity for preventive therapy, alongside curative solutions.

Based on the known underlying mechanism of AGIHL, compounds exhibiting neuroprotective, antioxidant, anti-apoptotic or immunomodulatory properties may help to preserve the hearing function during AG treatment. Furthermore, compounds that boost the function of DAergic neurotransmission in the endogenous protective LOC efferent system might also be protective. Screening of 640 compounds in an FDA-approved drug library suggested that DA-modulating drugs bear protective effects against ototoxic aminoglycosides and the antitumor drug cisplatin [65].

Noise trauma, ischemia or AGs cause excessive Glu release from IHCs that leads to excitotoxic damage at the synapses between IHCs and spiral ganglion neurons (SGCs), resulting in SGC dysfunction or ultimately death [7, 16, 30]. DA, released from LOC efferents, protects the IHC-SGC synapses and the SGCs by reducing Glu-mediated excitotoxicity [33, 55, 66]. We have already shown that 5-HT_6/7_ antagonists [67], group II mGluR ligands [68], selective NMDA receptor ligands [31] and D_2_ DA receptor antagonists [32] provide new possibilities for the enhancement of DA release from the LOC terminals in the cochlea [33].

Various therapeutic strategies have been investigated against SNHLs, including antioxidants, ROS/RNS scavengers, apoptosis inhibitors, neuroprotective agents, anti-inflammatory drugs, neurotrophic factors, and gene therapy [69]. However, achieving consistent clinical outcomes remains challenging. Several studies, investigating stem cell-based treatments, have indicated significant improvements in hearing thresholds, while other studies have reported no effect [70, 71]. Adeno-associated virus (AAV)-mediated otoferlin (OTOF) gene therapy has been identified as a potential treatment for the hereditary autosomal recessive deafness 9 (DFNB9), caused by the pathogenic mutations in the OTOF gene [72]. Despite its promise, gene therapy faces challenges, such as immune responses, off-target cell transduction and viral infection [73, 74]. There are several potential drug candidates specifically for the treatment of AG-ototoxicity in the preclinical phase. The most promising and important targets are the mechanoelectrical transducer (MET) channel, oxidative stress, apoptosis and autophagy [14, 75, 76]. According to clinicaltrials.org and a recent review [69] 32 compounds have reached clinical trials for SNHL, including 22 small molecules, six biological agents and four gene therapies. Among these, three small molecules (Ebselen, N-acetylcysteine (NAC) and ORC-13661) are being investigated for the prevention or treatment of AG-induced ototoxicity. ORC-13661 targets the entry routes of AG, NAC targets the oxidative stress pathway and Ebselen (SPI-1005) targets the apoptosis pathway [76]. Trials of Ebselen and NAC for AGIHL have already been completed without published results, while the ORC-13661 trial is not yet recruiting. Small molecules with better pharmacokinetics and more economical sustainability might offer a more comprehensive and better strategy for the diverse forms of hearing loss (HL).

Despite the advances in SNHL research, no pharmaceutical agent has been authorised for treating drug-induced SNHL until 2022. In that year and 2023, the FDA and European Medicines Agency (EMA) approved sodium thiosulfate (PEDMARK® and Pedmarqsi™, respectively) therapy for reducing cisplatin ototoxicity in children suffering from non-metastatic solid tumours [77–80]. This is a repositioned therapeutic indication of sodium thiosulfate, partly substantiated by its anti-inflammatory and antioxidant properties [81].

Our previous work has highlighted the otoprotective potential of the irreversible MAO-B inhibitor rasagiline in AGIHL [34]. More recently, selegiline, another FDA-approved irreversible MAO-B inhibitor with similar chemical structure and pharmacodynamic properties, was found to alleviate age-related hearing loss in our mouse model [35]. Because of the similarity in the pathomechanism of different SNHLs and the known DAergic [82], anti-apoptotic [24], anti-oxidant [25], neuroprotective [26] and immunmodulatory actions [27] of selegiline, we initiated a study on the effects of selegiline and its analogues on DA release from LOC terminals in the mouse cochlea and in an AGIHL mouse model. Although selegiline’s potential for otoprotection in mammals has been patented (US5561163, EP 0 831 798 B1), it was based on the generalisation of the result of a moderately controlled study on old outpatient dogs whose age-related hearing loss was assessed by inadequate behavioural responses to sounds such as commands and owners’ acknowledgements. They simply did not report the mechanism by which selegiline works, either.

Similar cochlear anatomy, physiology and ototoxicity-related HL (typically affecting the highest frequency hearing first) makes the mouse an experimental subject of human AGIHL with reliable translational power [12, 56]. These preclinical models provide the possibility of using a control and treatment groups experimental design, drug interventions, induction of AGIHL and the objective measurement of the hearing function.

We have shown that the known DA release-enhancing effect of selegiline [83, 84] is also exerted in the cochlea. It increased dose-dependently the nerve impulse propagation-evoked DA release from LOC terminals, that supposed to exert an otoprotective effect [38–41]. Based on our data, the potentiating effect of selegiline was dependent dominantly on the inhibition of MAO [84] and DA reuptake [58, 84, 85]. The increase in the vesicular DA pool, due to MAO inhibition, provides an opportunity for the amplification of the release of DA by vesicular exocytosis. Reuptake inhibition further enhances the rise in synaptic DA levels, that is why the pre-inhibition of the uptake molecules with nomifensine or low temperature reduced the effect of selegiline on electric stimulation-evoked release of DA. While this latter result might also suggest the involvement of transporter reversal, our findings do not support a dominant role for this mechanism or for the weak-base effect of protonated selegiline [86]. This is because resting release of DA was not enhanced—as would be expected if reverse transport [87] from an elevated cytosolic DA pool were occurring—and, in contrast, action potential-evoked DA release increased with rising concentrations of selegiline. Notably, if selegiline were reducing the synaptic vesicle pH gradient through its weak-base properties, this would promote the redistribution of vesicular DA to the cytosol, thereby decreasing vesicular content and consequently lowering evoked DA release [88], which is the opposite of what we observed.

Selegiline loses its selectivity as an inhibitor of MAO-B with increasing doses and—as it is an irreversible inhibitor—with an increasing number of administrations, i.e. during chronic treatment. Ekstedt et al. showed that 1 mg/kg daily s.c. injection for 21 days causes a ~ 70% MAO-A inhibition in rat brain [89]. Lamensdorf et al. found that treating rats for 21 days with 0.25 mg/kg s.c selegiline resulted in a 35–40% MAO-A inhibition [90]. Although there are significantly less study on mice, the loss of selectivity is also there in the mouse. Heikkila et al. demonstrated that long-term administration of doses above 2 mg/kg in mice are associated with diminished MAO-B specificity and increasing inhibition of MAO-A [91]. Therefore, the doses we used are probably at and above the borderline of losing MAO-B selectivity. A dose of 0.5 mg/kg is presumably still considered to be a predominantly selective dose in the cochlea, whereas doses of 3 and 6 mg/kg could also cause significant MAO-A inhibition. This may have played a role in the greater protective effect of the higher doses. It is worth mentioning that both MAO-A and MAO-B genes are expressed in the mouse cochlea (https://www.informatics.jax.org/gxd; MGI ID: 96915 and 96916) [92] and selegiline penetrates the cochlea very well, with a similar level of penetration to that in the brain [93].

The DA release enhancer effect of selegiline can mitigate the excitotoxic damage of SGCs, when LOC efferents are activated for hearing protection, i.e., selegiline amplifies the endogenous defence mechanism in AGIHL. Four chemically close analogues of selegiline were also tested on DA release, but only one (SR60490A) had an effect similar to selegiline. This means that even a minimal change in the chemical structure, while maintaining the propargylamine moiety, is sufficient to lose the potentiating effect on the action potential-evoked DA release.

Based on the in vitro findings, we further investigated the potential otoprotective effects of selegiline and SR60490A in vivo. Kanamycin-induced hearing loss (an AGIHL) was attenuated by the administration of a single daily injection of selegiline or SR60490A in a dose-dependent manner, with a similar action profile. The most pronounced effect was observed at 16 kHz, within the optimal hearing sensitivity range of mice (15–20 kHz; [94]), which is equivalent to the 1–4 kHz human optimum of hearing sensitivity. Presumably, all the aforementioned properties of selegiline, the antioxidant, anti-inflammatory, neuroprotective, antiapoptotic and DAergic enhancer effects contributed to its protective action. It has been reported that nuclear factor erythroid 2-related factor 2 (Nrf2) contributes to preventing gentamicin-induced cochlear injury and its activation could be protective against ototoxic agents [95]. Selegiline enhances the Nrf2 activity by increasing its nuclear translocation and its binding to the antioxidant response element (ARE) [96], thereby supporting the protective effect of this transcription factor. Additionally, it has been observed that nucleotide-binding oligomerisation domain-, leucine-rich repeat-, and pyrin domain-containing protein 3 (NLRP3)-inflammasome is involved in the development of kanamycin-related hearing loss. Blocking the activation of NLRP3-inflammasome by oridonin alleviated hearing impairment and hair cell death in mice [97]. Based on the knowledge that selegiline potentiates DA release and DA blocks NLRP3 inflammasome activation through the D_1_ receptor [98], selegiline could contribute to the inhibition of NLRP3-inflammasome and mitigate its harmful effects on hearing [99, 100]. In several studies, increased levels of brain-derived neurotrophic factor (BDNF), neurotrophin-3 (NT-3) and B-cell lymphoma 2 (Bcl-2) alleviated cochlear injury caused by AGs [101–103]. Selegiline could activate the intracellular signalling pathways of these neurotrophic and anti-apoptotic factors, thereby protecting cochlear cells from ototoxicity [104, 105].

To clarify the underlying mechanism of the otoprotective effect of selegiline, the effect of compounds with similar or overlapping chemical structures and pharmacodynamic properties on hearing has been investigated. Regarding hearing protection, safinamide has several beneficial properties such as antioxidant [45], neuroprotective [44] and anti-inflammatory effects [46]. Nevertheless, it did not affect kanamycin-induced hearing loss (AGIHL) in our mouse model. Unlike selegiline, safinamide is an α-aminoamide derivative and its MAO-B inhibitory effect is reversible [43]. In the dose we used, it preserves MAO-B selectivity, which may be another reason for its ineffectiveness. Although LJP-1207 is not recommended for prolonged administration due to its hydrazine moiety [49, 106], it is suitable to represent the impact of SSAO/VAP1 inhibitors on hearing. Like safinamide, the non-propargylamine LJP-1207 did not influence kanamycin-induced hearing impairment (AGIHL) even though it has anti-inflammatory effects [107] and as an SSAO/VAP1 inhibitor, it could alleviate oxidative stress and neuronal damage, as well [47, 49, 50]. In contrast to the other compounds tested in our AGIHL model, LJP-1207 has minimal inhibitory effect on MAO enzymes. Its potency is substantially higher for SSAO/VAP1, making it a selective SSAO/VAP1 inhibitor [47]. It has been reported that MAO-B activity produces H_2_O_2_ [22], in addition, may act as a suppressor of the constitutional expression of pro-survival genes, e.g. Bcl-2 and neurotrophic factors [108]. Based on these findings, the reversible and short-lasting inhibition of MAO-B by safinamide administration, the absence of MAO inhibition during LJP-1207 treatment or the lack of a propargylamine moiety in both molecules, which have intrinsic neuroprotective activity [109, 110], could contribute to their ineffectiveness in our AGIHL model.

Compared to rasagiline, which has previously been shown to be otoprotective against AGIHL in 6 mg/kg dose at 16 kHz [34], selegiline and SR60490A exhibited a broader protective frequency range (click, 4, 8, and 16 kHz) and were effective at multiple doses (0.5, 3, and 6 mg/kg). This greater protective action of selegiline and SR60490A, in contrast to rasagiline, may be explained by differences in their DA-releasing effect in the cochlea. Although rasagiline enhanced the stimulation-evoked release of DA (FRS_2_/FRS_1_ = 1.82 ± 0.25 at 30 µM, and 2.04 ± 0.24 at 100 µM, respectively; [34]), the increase was more pronounced during both selegiline (FRS_2_/FRS_1_ = 2.31 ± 0.42 at 30 µM, and 4.16 ± 0.51 at 100 µM) and SR60490A (FRS_2_/FRS_1_ = 2.82 ± 0.28 at 30 µM) administration. The difference may originate from the distinct impact of selegiline on the trace amine-associated receptor 1 (TAAR1). Unlike rasagiline, which is suggested to antagonise TAAR1, selegiline activates TAAR1, which is supposed to increase the action potential evoked DA release [42]. Furthermore, TAAR1 is also involved in neuroprotection [111], and immunomodulation [112, 113], and contributes to synaptic plasticity [114], processes that may contribute to the protective effect of selegiline in AGIHL.

According to the FDA guidance [115] for mouse-to-human dose conversion, the 6 mg/kg protective dose of selegiline in mice equates to an approximate 34 mg/day human equivalent dose. The use of a higher-than-human antiparkinsonian dose (5–10 mg/day) of selegiline raises the possibility of enhanced psychomotor activity, because selegiline is metabolised to *R-*methamphetamine and *R-*amphetamine [22]. Although the (*S*)-enantiomers of methamphetamine and amphetamine are the more potent psychoactive agents [116, 117], we investigated the possible motor activity-enhancing, anxiogenic and anorectic effects of selegiline [118, 119]. However, it did not modify, even at the highest dose used, the behaviour of the animals and their weight gain also remained within the normal range. Furthermore, we have not observed any other behavioural changes during the chronic treatment that could have been triggered by an unwanted side effect of selegiline on hearing. The chronic treatment with selegiline or SR60490A alone did not influence the hearing threshold at all, at any measured frequency.

There is no effective pharmacotherapy for SNHLs. The disease, including AGIHL, affects millions of people worldwide and generates an unmet clinical need for both prevention and treatment. We demonstrated that administration of selegiline, a registered, irreversible MAO-B inhibitor anti-Parkinson medication with propargylamine moiety in its chemical structure, has attenuated the kanamycin-induced hearing loss (an AGIHL) in mice. The protection of the hearing function could be explained by a combined antioxidant, neuroprotective and anti-inflammatory effect complemented by the enhancement of the action of an endogenous hearing protection mechanism via DA release from LOC efferents, the effector arm of a feedback loop, that attenuates the excitotoxic overactivation of the primary auditory neurons and their synapses with the IHCs. One of the very close chemical analogues of selegiline, SR60490A, produced the same effect in both potentiating the action potential-evoked release of DA from LOC efferents and the mitigation of hearing impairment induced by AG. In contrast, safinamide, a reversible inhibitor of MAO-B enzyme and Glu release and LJP-1207, a selective SSAO inhibitor with antioxidant and anti-inflammatory effects, and as such, compounds with considerable pharmacodynamic overlap with selegiline, but different, non-propargylamine structures, failed to be otoprotective in AGIHL. This suggests that the propargylamine moiety, with its intrinsic neuroprotective property, and the activation of the LOC DA system via a potent MAO enzyme and DA uptake inhibition might be necessary components of the multitarget otoprotective action. It also highlights the fact that not all compounds that are chemically and/or pharmacodynamically very similar to an otoprotective compound will have a positive effect on AGIHL. Minimal differences matter. The complex, multifactorial pathomechanism of AGIHL most likely requires drugs owing to an optimal multitarget profile for effective therapy. Selegiline, as an FDA-approved drug with a favourable drug safety profile, might be a promising candidate for repositioning to treat AGIHL or other forms of SNHLs.

## Data Availability

No datasets were generated or analysed during the current study.
